# Patient Perspectives on Engagement in Recovery after Hip Fracture: A Qualitative Study

**DOI:** 10.1155/2017/2171865

**Published:** 2017-03-20

**Authors:** Joanie Sims-Gould, Sarah Stott-Eveneshen, Lena Fleig, Megan McAllister, Maureen C. Ashe

**Affiliations:** ^1^Centre for Hip Health and Mobility, Robert H.N. Ho Research Centre, 2635 Laurel Street, Vancouver, BC, Canada V5Z 1M9; ^2^Department of Family Practice, University of British Columbia, Vancouver, BC, Canada; ^3^Freie Universität Berlin, AB Gesundheitspsychologie, Habelschwerdter Allee 45, PF 10, 14195 Berlin, Germany

## Abstract

*Purpose. *To understand older adults' engagement in their recovery experience and rehabilitation after a fall-related hip fracture.* Method. *50 community-dwelling older adults recovering from a recent (3–12 months) hip fracture (32 women, 18 men) participated in telephone interviews using a semistructured format at 6 and 12 months after recruitment into the study. Interviews were conducted as part of a mixed-methods study designed to test the effect of a postoperative hip fracture management program (B4 Clinic).* Results. *Three substantive themes were identified in the qualitative data: (1) managing expectations; (2) engaging in physical activity; and (3) there is life after fracture. Participants shared valuable insight into how their expectations for their recovery period compared to their lived experience and the role of physical activity in their ability to return to their prefracture activities.* Conclusions. *Our findings reflect older adults' expectations for recovery from hip fracture. Encouraging engagement in rehabilitative exercises and addressing expectations prior to hospital discharge may improve patients' adherence to rehabilitation programs, functional outcomes, and postoperative quality of life. Implications for rehabilitation include the necessity for early and ongoing engagement of rehabilitation professionals.

## 1. Introduction

A universal finding in health care research is that patients report difficulty navigating health care systems and that a major factor is a lack of information at the right time, in the right place, from the right person [1, 2]. The Change Foundation (2008) [3] reported that over 40% of surveyed Canadian patients discharged from hospital either did not know who to contact with questions about their condition or treatment or did not receive instructions about symptoms to watch or had no follow-up arrangements made by their hospital. Information is not often shared between health care providers and their patients [4, 5].

Patient engagement is a goal for health care systems, yet full participation in care requires a multitude of actions to ensure that one has the information they need and can experience the highest form of patient engagement: empowerment [6]. Patient engagement is defined by the Centre for Advancing Health [7] as the actions individuals must take to obtain the greatest benefit from the health care services available to them and, as such, places an emphasis on the patients' role in their own care. Specifically, behaviors that contribute to patient engagement include patient participation in making decisions, asking questions, and seeking information. Patient engagement is important because patients who are prepared to manage their symptoms, engage in health promoting behaviors, engage in decision-making, engage with providers, understand quality of care, and navigate the health care system tend to have better health outcomes [6, 8–10]. Patient engagement is particularly important in those circumstances where care needs are high and often complex like in the case of a hip fracture. This is particularly true for those who become patients very suddenly as is the case with those who fracture a hip. Not only must patients and their families become engaged in their care very quickly, they must maintain engagement over time and across changes to their health situation. Central to understanding engagement in care is an understanding of patient experiences [11]. Our research examines the experiences of patients after hip fracture. We focus specifically on patient's perceptions of their recovery experience and their engagement in rehabilitation after hip fracture.

## 2. Methodology

### 2.1. Design

This qualitative substudy was part of a larger single-centre randomized control trial (RCT) (Clinical Trials Registration NCT01254942) testing a specialized postoperative hip fracture management program (B4 Clinic) with usual care compared with only usual care (UC) [10]. The primary aim of the study was to test the effect of the B4 Clinic on mobility (operationalized as the Short Physical Performance Battery). For both the RCT and the qualitative substudy we obtained university and hospital ethics approval, and all study participants provided informed written consent. All participants randomized to the intervention group received usual care as above, plus a comprehensive assessment by a geriatrician based on the clinical practice guidelines from the American and British Geriatrics societies. In addition, all participants were assessed by a physiotherapist (PT) and occupational therapist (OT). The geriatrician referred participants to other health professionals (e.g., continence nurse) and/or specialists (ophthalmologist) as deemed appropriate by the initial assessment. As needed, participants attended PT and/or OT sessions on an outpatient basis and/or were given a home exercise program.

### 2.2. Participants

Participants were community-dwelling older adults age 65 years and older living in Metro Vancouver, who had a hip fracture (proximal, middle, or distal femur) in the 12 months before data collection. All study participants, from both the intervention and control groups, were invited to participate in this qualitative substudy. Participant's demographics are described in Table 1.

### 2.3. Data Collection

Three trained interviewers completed over-the-phone interviews of approximately 20 minutes (range 5–30 minutes) with 50 participants at 6 months (mid-point) and 12 months (final) after recruitment. Interviewers took extensive notes during the interviews. These notes were then transcribed by the interviewer verbatim. At each point of data collection, 48 participants agreed to complete the interview, with 45 participants participating in both interviews. Those who declined to participate in both qualitative interviews are not included in the described results. The questions asked at each interview are outlined in Table 2. As per our ethics all of our data has been deidentified and pseudonyms have been used.

### 2.4. Data Analysis

All data were deidentified and organized into a question-and-answer format prior to importing into NVivo 10®, a computer-assisted data analysis program (QSR International, 2015). A deductive approach was used for initial structural coding to create a broad coding framework a priori based on the aim of each interview question, such as participants' expectations and goals for their recovery, their ability to resume their prefracture activities, and their reasons for joining in the study. Following this first cycle of coding, we used an inductive approach to conduct a second cycle of descriptive coding and a third cycle of line-by-line coding to ensure the assigned codes (i.e., short, descriptive headings assigned to a portion of a transcript) were representative of participant responses [11]. We recorded notes on the procedures and decisions made during data analysis and reflections on emerging themes in a reflexive journal. The study team met to discuss the coding and preliminary findings after each cycle of coding. These discussions informed the following cycle of coding and generated questions to be examined using investigative queries in NVivo 10, such as, “What factors supported participants who reached their recovery goals?” and “What were the difficulties participants encountered during their recovery?” The use of queries was valuable for analyzing the intersections between codes, frequency of responses, and the role of select variables in participants' recovery experiences. The final codebook and the results of these queries were discussed among the research team until consensus was met on the substantive themes in the data. The researchers are in agreement that saturation was obtained in the data, as no new themes were emerging in the last interviews.

## 3. Results

Three main themes were found to describe the recovery experience of patients: (1) managing expectations, (2) engaging in physical activity, and (3) there is life after fracture. While distinct, the themes share important intersections that cross the patient experience of recovering from hip fracture, from preoperative expectations for their recovery to the processes that support or hinder engagement in rehabilitation.

### 3.1. Managing Expectations

Participants were asked to describe their expectations for their recovery from hip fracture. For 16%  (*n* = 8) of participants, the recovery period was what they had expected. However, for 62% of participants (*n* = 31) the recovery experience was not what they had expected, often because of the length of time required to recover and the onset of unexpected postoperative complications, described by 18%  (*n* = 9) of participants as being the most unanticipated event in their recovery. Most participants expected to be fully recovered within 6 months or less after their surgery for hip fracture, as told by their surgeon.

“The doctor said in 3 weeks I'd be feeling fine and in 6 weeks I'd be back to everything, and I'm not. It's a bit depressing. It's been a long time”.* Ella, female, aged 80.*

The source and type of information patients receive preoperatively has an important role in the formation of realistic expectations for their recovery. Those who had high levels of engagement and had sought the perspectives of friends and family members who had personal experience with postoperative recovery from hip fracture or read the advice of past patients on Internet message boards managed their expectations better than those who had not. One participant commented on not knowing what to expect preoperatively and the difficulty in obtaining enough information to manage their expectations. They indicated that “it is hard when information is scare [sic] or incorrect.”

“As a patient we are at huge disadvantage because we don't know what questions to ask. … I didn't know … to ask what side effects or residual problems there might be. And I don't know how to get around it, because the surgeons are excellent and capably answer all the questions I did ask, but I can't expect them to tell me everything they know. I tried googling information, but there wasn't the type of discussion I was looking for online. In speaking with another person who had a hip fracture, I recognize that the experience can be quite different”.* Robert, male, aged 87.*

Holding unrealistic expectations increases the risk of feeling disappointed and dissatisfied when the level of functional ability recovered does not meet the expectations initially set.

“I thought I would be 100% or more or less as I was before … I was very optimistic … It is taking much longer than I thought. I guess you could say I'm disappointed. But I look at the stats for people like me and I think I'm doing well. I can live with it when I look at what happens to other people”.* Margaret, female, aged 81.*

For three participants, not meeting their expectations discouraged them from continuing to engage in rehabilitative exercises, feeling as though little to no progress was being made.

### 3.2. Engaging in Physical Activity

The theme of “engaging in physical activity” describes the role of staying engaged and active after hip fracture surgery. For several participants, staying active was a means of regaining autonomy and taking control over the recovery by engaging in behaviors they believed/expected directly contributed to their recovery, such as walking outside and attending exercise classes or physiotherapy sessions. Several participants attributed their ability to return to their prefracture activities to their self-determined motivation; for example, Doreen stated, “because I wanted to” and Ester indicated: “I was determined I would.”

For some, their continued belief that you have to keep moving during recovery from hip fracture stems from their optimism and emphasis on keeping a positive attitude (e.g., anticipation of future positive outcomes) as well as their confidence to persevere in the face of challenges, such as pain or slow progress.

“I would say the attitude mentally is very important. When talking to other people about the study and why I am involved, some don't want to … they say it hurts, but I don't say that, I just work at it. It's what the physio says is for your own good, and that positive mental attitude is important”.* Ethel, female, aged 90.*

Others reported disengaging from their recovery and ceasing continuation of their exercises after months of not seeing progress and feeling discouraged and dissatisfied. At 6 months after recruitment into the study, one participant commented,

“I wouldn't say I have a real goal [for recovery] because it [mobility] isn't going to change”.* Clara, female, aged 80.*

Clara's comment is a stark contrast to those made by Doreen and Esther, reflecting her perceived lack of control over her ability to change her functional outcome. Those who participated in a progressive exercise program, either through the B4 Clinic or their own home exercises, commented on how doing daily exercises provided a means for them to assess their progress throughout the recovery period:

“I can see I still have a long way to go. It's good, it [exercises] shows me how I can improve”.* Julius, male, aged 93.*

For most participants, exercise and other physical activities were an important part of their recovery. Within the intervention group, this was expressed by 60%  (*n* = 15) of participants compared to 52%  (*n* = 13) of those in the control group. While the control group did not have any formal activity program during the study, many still engaged in physical activity through their own physiotherapy or home exercise program. For several participants, keeping active was an important factor for pain management.

“When I don't do exercises, my hip hurts and I say to myself it serves you right, you're not doing your exercises”.* Vera, female, aged 90.*

Among participants who were not actively involved in exercises, some would comment that they knew they should be doing more or expressed an intention to start getting involved in activities pending the outcome of another ailment or condition in their life. Reasons for not exercising included medication side effects, complications from other health conditions, pain, lethargy, poor weather, and a lack of rehabilitation support (i.e., wanting more physiotherapy services than was offered to them). Several participants expressed feeling that they needed to wait for their body to heal more before they could safely exercise and were, therefore, less likely to engage in exercise activities.

### 3.3. There Is Life after Fracture

This theme captures participants' perspectives on their return to their prefracture life and activities. Participants predominantly fell into two categories of recovery: those who resumed their prefracture activities or an adapted version of them (*N* = 33) and those who felt they will never recover to their prefracture functional ability (*N* = 12). Those who resumed their prefracture activities were more likely to have more engagement in their postfracture care and rehabilitation. This perspective led them to take up participation in new activities (e.g., physiotherapy, home exercise program, or regular walking outside) or modify their prefracture activities to accommodate their changed level of functional capacity (i.e., flexible goal adjustment).

“… there was some pain during the 6 months before the screws were removed, but it was not an impediment [to my activity]. I found ways to hike without pain. [What was that?] Oh, well when I went up steep inclines I would walk on my toes”.* Robert, male, aged 87.*

Those who described a return to their prefracture activities often emphasized the importance of keeping a positive perspective throughout their recovery to mitigate feelings of disappointment or depression over a slow progression. This was implemented in their daily lives by consciously appreciating the small gains made and creating a flexible timeline for their recovery goals.

“I've changed a bit what I do, and I guess I do it all but much slower. I do all other things I did before … I walked a lot better [before]. Now I don't know if I will ever be able to, I concentrate and it is a chore”.* Margaret, female, aged 81.*

When participants discussed their progress made, it was often qualified that while they are better than they were at the beginning of their recovery period, they are still not back to where they were before hip fracture.

“Still working towards better balance, better strength, better speed. I tell other people about it and they say I am doing well and perhaps I am for my age, but I am not where I was before. … Biking is better and back country skiing is alright, but I'm not as graceful going down stairs – I hold on tight when no one is there and am hesitant. And I don't thinks it's just mental…. I'm more fatigued, I think it might have to do with aerobic strength and endurance. … I'm just not where I used to be”.* Ella, female, aged 80.*

Some participants described their recovery in terms of a percentage of what their abilities were before hip fracture.

“I had quite extensive physiotherapy … after my hip [surgery] and I came out feeling 95%”.* Martin, male, aged 80.*

Participants were asked at their final interview if they had achieved the recovery goals that they set for themselves. One participant responded that, “there is life after fracture,” and although she did not meet her mobility goals, she sees her recovery as a long-term journey requiring long-term engagement in her rehabilitation activities (e.g., home exercise routine, regularly walking outdoors). For participants who described having had a history of being physically active, this perspective reflects a continuation of their prefracture activities. For others, particularly those over age 85, “life after fracture” is obtained through the ability to engage in activities that bring enjoyment to their life, such as visiting with family members (i.e., social participation). Among all participants, those over age 85 (*N* = 12) more frequently expressed feelings of acceptance over their loss of functional ability than younger participants. This was often attributed to the progression of other chronic conditions or their age-related expectations. To pursue and maintain personally relevant activity goals, individuals appeared to adjust their goals in response to (perceived) losses in their functional ability.

“I'm realistic about my goals. I know at my age I can't go back to all the activities I used to do”.* Stanley, male, aged 93.*

Participants also discussed the importance of social support during their postoperative recovery period. For those living with a spouse or other family member, family was often described as being instrumental for support with daily activities and encouragement to engage in rehabilitative exercises. While caring health care staff was important to most participants, those who lived alone express the greatest appreciation for this source of support. Study-related monthly phone calls to check in on participants' progress was also described as an important source of social support and motivation among some participants.

“I felt safe and secure knowing that you and other [research assistant] girls were following my progress and that if anything went very wrong I could call – I don't' know if it would do any good, but it gave me a good feeling”.* Dorothy, female, aged 93.*

## 4. Discussion

Hip fracture is a serious life event that has important implications for older adults' personal identity [12], mortality risk [13], and collective health care costs [14]. The theme of “managing expectations” highlights participants' expectations for their recovery and their lived experiences. Participants often described being told by their surgeon to expect a recovery period of 6 months before being able to return to their usual, prefracture activities. It is common for people to underestimate the length of time required to recover from hip fracture [15], particularly when the risk of complications is not taken into account [16]. While some studies suggest that the majority of recovery occurs in the first 6 months after surgery [16], others suggest that recovery can take up to a year or longer [12], with up to a 25% loss of prefracture functional ability remaining for some patients [15, 17]. In one study, only 39% of patients with hip fracture were fully back to their prefracture levels of mobility after one year [16]. Rehabilitation outcomes may be even worse among patients who reside in long-term care, as they often do not obtain the same level of access to rehabilitation services as those residing in the community [18].

As a result, many patients go into their recovery uncertain or misinformed about the length of time required to recover and the activities that would best support their recovery. This issue is compounded by a lack of information provided to patients [19], either as a result of limited resources or a lack of understanding of what is needed by patients for recovery or lack of patient engagement (or some combination). As demonstrated in our results misinformation can further exacerbate disengagement from one's recovery [20, 21]. Providing patients with a more realistic timeline for their recovery has its difficulties given the heterogeneity of those experiencing hip fracture.

This issue highlights a need for increased information sharing and preoperative counselling on the aims of the surgical intervention, risk of complications, expected length of recovery given patients' fracture type, and the importance of early mobilisation and integration of balance and strength exercises for recovery [15]. The use of a preoperative classification system has been suggested as a way to assess patients' characteristics as a means of supporting practitioners to obtain a more personalized projection of the expected recovery timeline for their patients; therefore improving their ability to engage patients appropriate and plan rehabilitative services accordingly [22]. This approach serves to manage patients' expectations and empower them to increase adherence to a rehabilitation program, even when progress may be slower than anticipated [19, 23]. An enhanced understanding of the patient experience may further increase the capacity of health care practitioners to address questions patients do not know to ask, increasing the likelihood that the information they receive is accurate and applicable to their specific condition and fracture.

Staying active and engaging in balance and strength exercise is one of the most effective postoperative measures for patients' return to their prefracture level of functional ability and independent ambulation [20, 24]. Engaging in physical activity was discussed by participants, who emphasized the importance of rehabilitation services, such as physiotherapy, in not only teaching patients what exercises they can do to improve their functional outcomes, but also in motivating them to keep up with their exercises if progress is not initially realized. One participant in our study described the advice of her physician to simply walk more after she asked about participating in physiotherapy; however, exercises prescribed by physiotherapists can better utilize the movements required to perform daily life activities and, as such, better prepare patients to return to these activities than solely walking [21].

Consistent with previous research, the most commonly reported barriers by study participants for engaging in physical activity were pain from complications during recovery [21, 25], lack of information on what they could physically do without hindering their recovery [26], and low motivation or acceptance of their changed level of functional ability [25, 27]. Intrapersonal, psychosocial factors (e.g., positive attitudes, optimism, expectations, motivation, self-efficacy, and outcome experience) and the reception of social support from family, friends, and health care providers can also play an important facilitating role in recovery [12, 21].

In our study, three participants reported discontinuing their exercise activities after not seeing the progress they had expected by 6 months after their surgical intervention. This is in line with previous behavior change theory and research which suggests that (unrealistic) expectations that do not match lived experiences result in dissatisfaction and ultimately disengagement (e.g., [28]). Other participants (*N* = 6) continued with their prescribed physiotherapy but expressed feeling that the exercises were not making a difference to their recovery. For these participants, their disengagement from exercise may not only reflect a lack satisfaction with expected recovery but also a lack of perceived control over their ability to effect change on their functional outcome. However, the majority of participants described seeing noticeable gains in their functional ability over time as a result of their continued participation in exercise activities, such as those prescribed by their physiotherapist. For these participants, exercise had a vital role in their recovery, with many participants expressing their appreciation for the physiotherapy services received.

For those participants who attributed their recovery to their own desire to participate in physical activity which served as a way for them to exert control over the recovery of their functional ability. Comments made by these participants also reflect high level of perceived confidence in their ability (i.e., self-efficacy) to participate in exercise programs and progress in their exercises as they advance in their recovery. Studies show that improvements in patients' level of self-efficacy can effectively increase adherence to exercise activities and improve functional outcomes [8, 29–31], particularly in the first 6 months after surgical intervention [32, 33]. Adherence further increases when exercise is integrated into patients' daily routines [21], and improvements to ambulation and functional ability begin to be realized [21, 28]. Besides self-efficacy, attributing progress in recovery to one's own efforts (e.g., through continued engagement in activity)/having an internal locus of control has been associated with greater independence in performing activities of daily living and lower levels of disability [34].

Studies have shown that between 6 and 12 months after hip fracture, older adults are more likely to experience a decline in their adherence to exercise programs as a result of increased fear of falling, experiencing pain attributed to the onset of complications or new health problems [31], low self-efficacy, or the lack of accountability from the cessation of group or in-home exercise sessions [35]. Therefore, it is important that rehabilitation interventions include components that postoperatively address patients' level of self-efficacy by continued engagement with rehabilitation professionals.

The research on the appropriate location and length of delivery of a rehabilitation program to older adults recovering from hip fracture is mixed. While some studies suggest that short-term programs reduce the risk of dependency on health care professionals and better facilitate functional independence [36] and increase self-efficacy [20], others suggest that functional gains made during the initial recovery period may be lost following program cessation [37], making an extended outpatient rehabilitation program with progressive training more ideal [32]. Participation in extended outpatient programs for at least 6 months has been found to offer measureable improvements in patients' muscle strength, balance, and gait speed, compared to participation in only a low-intensity home exercise program [32]. When measured at one year after hip fracture, those who participated in extended outpatient rehabilitation programs also had a greater level of perceived health [32], walking confidence [33], self-efficacy [33], and quality of life [38], likely a result of the gains made in their functional ability during their extended rehabilitation [39].

Some researchers have argued that longer-term programs are unnecessary given that most improvements to functional ability are made in the initial 4–6 months after hip fracture before plateauing [16], to which others assert that this can be overcome by increasing the intensity of exercise therapy and targeting improvements to patient balance, knee strength, and gait speed throughout the recovery period [24]. It is well understood that the integration of ambulatory and progressive rehabilitation programming into a postfracture care plan is necessary for improving patient strength, balance, and functional independence in completing activities of daily living [16, 33]. Programs that utilize elements of both types of rehabilitation programs, such as coupling the delivery of a short-term, inpatient program with a long-term, progressive outpatient program for up to 4–6 months with an integrated balance, and strength component, may offset declines in patient adherence and produce the best functional outcomes [16, 20, 24, 40, 41]. We support a model that includes sustained engagement.

The final theme in our study highlighted participant's view of life after hip fracture. Three participants in our study described feeling depressed after their hip fracture, which is not uncommon for patients recovering from such a health event [42]. It is also common for patients to have a heightened fear of falling after hip fracture [36] which may hinder their participation in physical activities that would support their recovery (e.g., walking outdoors, balance and strength activities) [37, 38]. By limiting their engagement in these activities, patients place themselves at greater risk of a subsequent fracture [24] for at least 10 years after their initial fracture [43].

Factors found to support or enhance recovery from hip fracture include staying motivated and determined to participate in exercise activities (in hospital and after discharge at home), self-regulatory strategies such as goal-setting and planning, social support, positive attitude, participation in health promoting behaviors (e.g., good nutrition, regular exercise, and taking appropriate medications), and a supportive physical environment conducive of physical activity (e.g., a walkable neighborhood) [44]. Receiving good quality care from health care providers (e.g., surgeon, physiotherapist, and family physician) can also affect patients' recovery. When care providers operate with a positive and friendly demeanor and provide adequate communication of information about the recovery process and address patient questions, recovery outcomes may improve [44].

Previous research has shown that patients with a more positive outlook on their recovery tend to show more signs of resiliency [45] and have better functional outcomes [46]. In our study, some participants attributed their positive attitude to the gains made in their recovery. It may be that those with a more positive perspective on their recovery experience are more likely to engage the rehabilitation supports available to them and keep up with their exercise activities for a longer period of time than those who get discouraged if not fully recovered after doing their exercises activities [47]. In our study, having social support through the encouragement of family members and research staff helped facilitate this positive thinking and keep them motivated to continue the rehabilitation journey.

Four participants who had not reached their original recovery goals in our study discussed the importance of not giving up, adjusting their goals, and continuing with the activities they could within their capacity (i.e., accommodative coping tendency, flexible goal adjustment in response to loss/physical constraints). This ability to accept their circumstances has been related to expressions of resilience in older adult participants in other qualitative studies [48, 49]. For others, their alignment with the theme, “there is life after fracture” is meant in the literal sense, as described by their appreciation for being alive after such as traumatic health event. Participants' discussion of their recovery from hip fracture as a long-term process highlights their continuation with life activities under the adoption of a new perspective. Studies have shown that life after fracture encapsulates a breadth of considerations that extend beyond the rehabilitation of independent ambulation and functional rehabilitation [12, 50]. Similar to findings of previous studies, adapting travel patterns, engaging in everyday activities, signing up for exercises classes at a local community center, and being more cognizant of fall risks are merely some of the ways that participants moved on with their life after fracture [12, 28].

*Study Limitations*. The aim of our study was to explore the patient experience of recovering from hip fracture among those who participated in the randomized controlled trial. While the themes identified in this study encapsulate the patient experience from the pre- to postoperative periods, several limitations have been identified. The first being that the interviews were not audio recorded. While vigorous notes and verbatim quotes were transcribed by the interviewers during the phone interviews, the transcripts do not reflect a verbatim account of participants' responses. This may have resulted in a recorder bias, where the decision by the interviewer as to what information is deemed important may affect what is recorded. The length of the interviews were also shorter than is typical for a semistructured interview and may reflect the timing of the phone call. Another limitation is that participants were overall a very physically active group. As such, their experience may not reflect the “typical” older adult undergoing surgery for hip fracture. However, similarities have been found between the themes identified in this study and those of other studies using qualitative methods to describe older adults' recovery from hip fracture [12, 51, 52]. These consistencies enhance the body of knowledge in this area and provide a stronger foundation from which to propose recommendations for practice. We also note that the study size of 50 participants at a single center and almost 2 : 1 ratio of women to men limits the generalizability of our findings.

## 5. Conclusion

Our study captured the experiences of a group of participants who were fairly active during their recovery from hip fracture. While the recovery period was not what participants had expected, most were able to return to some of their prefracture activities, albeit with the necessary adaptions made to the activity in order to do so. By managing expectations, keeping active, and maintaining participation in activities that bring quality to one's life (e.g., exercise, social participation) older adults can resume their life after hip fracture.

## Figures and Tables

**Table 1:** Frequencies of participant demographics (*n* = 50).

Sex	
Women	32 (64%)
Men	18 (36%)
Marital status	
Married	27 (54%)
Widowed	8 (16%)
Separated/divorced	5 (10%)
Single	10 (20%)
Age	
65–74 years	14 (28%)
75–84 years	22 (44%)
85+ years	14 (28%)
Living arrangement	
Living alone	21 (42%)
Living with someone (spouse, friend, or family member)	29 (58%)
Completed high school	
Yes	45 (90%)
Education	
Completed postsecondary	33 (66%)
Comorbidity (2+ chronic condition diagnoses)	
No chronic conditions	11 (22%)
2–4 chronic conditions^*∗*^	26 (52%)
5-6 chronic conditions	10 (20%)
7+ chronic conditions	3 (6%)

^*∗*^Common chronic conditions include (in order of prominence): arthritis, visual impairment, osteoporosis, asthma, COPD, angina, heart disease, neurological, stroke, vascular disease, diabetes, gastrointestinal, depression, anxiety, hearing, degenerative, and obesity.

**Table 2:** Interview guide questions at 6 and 12 months after study onset.

6 months	12 months
What was your original expectation of your hip fracture recovery process?	Why did you decide to join this study after you broke your hip?
Have you been able to resume all of your pre-fracture activities?	Have you been able to achieve your goals regarding your recovery and return to your pre-fracture activities?
Do you have any goals for returning to your usual activities?	What, if any, benefit did you get out of your involvement in the study?
Related to your participation in the study, what can we do better moving forward?	Related to your participation in the study, what could we have done better?
